# Role of socio-economic research in developing, delivering and scaling new crop varieties: the case of staple crop biofortification

**DOI:** 10.3389/fpls.2023.1099496

**Published:** 2023-07-03

**Authors:** Ekin Birol, Howarth E. Bouis

**Affiliations:** ^1^ Global Human Development Program, Edmund A. Walsh School of Foreign Service, Georgetown University, Washington, DC, United States; ^2^ International Food Policy Research Institute, HarvestPlus and Micronutrient Forum, Los Banos, Philippines

**Keywords:** biofortification, socio-economic research, cost-effectiveness, impact evaluation, consumer acceptance, farmer adoption, monitoring, program evaluation and learning

## Abstract

The CGIAR biofortification program, HarvestPlus, was founded with the aim of improving the quality of diets through micronutrient-dense varieties of staple food crops. Implemented in four phases – discovery, development, delivery and scaling – the program was designed to be interdisciplinary, with plant breeding R&D supported by nutrition and socio-economic research. This paper explains the need, use and usefulness of socio-economic research in each phase of the program. *Ex ante* and *ex post* benefit-cost analyses facilitated fundraising for initial biofortification R&D and implementation in each subsequent phase, as well as encouraged other public, private, and civil society and non-governmental organizations to take on and mainstream biofortification in their crop R&D, policies, and programs. Socio-economics research helped guide plant breeding by identifying priority micronutrient- crop- geography combinations for maximum impact. Health impacts of biofortification could be projected both by using empirical results obtained through randomized controlled bioefficacy trials conducted by nutritionists, and through farmer-adoption models estimating impact at scale. Farmer and consumer surveys and monitoring systems provided the underlying information for estimating farmer adoption models and helped understand input/output markets, farmer and consumer preferences, and additional opportunities and challenges –all of which informed crop breeding and delivery activities, while building the knowledge base for catalyzing the scaling of biofortification.

## 1 Introduction

A plaque in the lobby of FAO headquarters reads:

“In this building, 16th of October 1945, representatives of 44 nations met and established the FOOD AND AGRICULTURAL ORGANIZATION, first of the new United Nations Agencies. Thus, for the first time, nations organized to raise levels of nutrition and to improve production and distribution of food and agricultural products.”

The objective of improving human nutrition is mentioned first, and agricultural supply second. This priority was reversed by the early 1960’s over concerns with increasing global population and possible famine, propelling national agricultural policies and investments in national and international agricultural research centers to focus on crop development for productivity, i.e. yield. This priority on yield and productivity, has made economic sense in terms of higher quantity and hence higher value of production per amount of land, and has resulted in significant reductions in hunger and poverty. However, decades of deprioritization of nutrient content of crops, especially of staples, in plant breeding programs, have resulted in the density of minerals and vitamins declining over time as yields have increased (see e.g., Davis et al., 2004; Fan et al., 2008)

More recently public health and nutrition research has shown that this focus of the agricultural sector on yield and productivity alone also did not make as much economic sense as initially thought, since the unaccounted for, i.e., “hidden’’ costs of nutrient deficiencies, e.g., of reduced labor productivity and returns to education as well as poor health, were not only high but also intergenerational. These unaccounted costs – also known in economics as externalities - were found to be especially high (and often showing an increasing trend, as a result of factors such as climate change, covid and conflicts) among populations with staple crop-based diets as prevalent in rural areas of low- and middle-income countries (LMICs) (see e.g., Stein and Qaim, 2007; Global Panel, 2016; Ambikapathi et al., 2022).

Recognizing the potential economic value of breeding for nutrient density simultaneously with yield and productivity, the CGIAR established a “biofortification” program in 2003. Called HarvestPlus, the program was developed and implemented in close collaboration between CGIAR centers and national agricultural research systems in several LMICs. The main aim of this program was to test the concept of biofortification by answering three questions: (i) is it possible to biofortify, i.e., to breed nutrient-dense varieties of key staples in high-yielding backgrounds by using modern breeding techniques and the genetic material available in the CGIAR and other national and regional gene banks?; (ii) could these biofortified varieties be sufficiently dense in bioavailable minerals and vitamins so as to result in improved micronutrient deficiency status among consuming populations?; and (iii) are farmers and consumers willing to grow and consume these varieties?. As the growing research started to answer the first three questions in the affirmative for several micronutrient-crop-country combinations, a fourth question, namely how can biofortified varieties of crops be scaled up, was added. Even though plant breeding was at the heart of the program, HarvestPlus also aimed to deliver and eventually catalyze the scaling of these biofortified crops once they were released by national systems for planting.

The overall impact HarvestPlus program strived towards was alleviation of micro nutrient deficiencies – in particular vitamin A, iron, and zinc deficiencies – in rural areas of LMICs. This meant not only plant breeding research to develop the biofortified varieties, but also socio-economic research to ensure that these varieties are developed with the needs and preferences of end-users (i.e., farmers and consumers) in mind, and once released, they are adopted by farmers and eaten by consumers, and nutrition research to assess that once consumed these varieties reduced micronutrient deficiencies in target populations. These requirements for impact translated into a need for developing an interdisciplinary team, consisting of plant breeders, nutritionists, socio-economists, and additional disciplines such as farm extension, behavioral change, and communications specialists.

The aim of this article is to document and share lessons learned from the development and implementation of the socio-economic research agenda of the HarvestPlus program. Our hope is that the process HarvestPlus followed for intentional interdisciplinarity in general, and the socio-economic research conducted to inform the development, delivery and scaling of biofortified varieties of crops in particular, will be useful for/used by other plant breeding initiatives that aim to make impact at scale.

**Figure 1:**
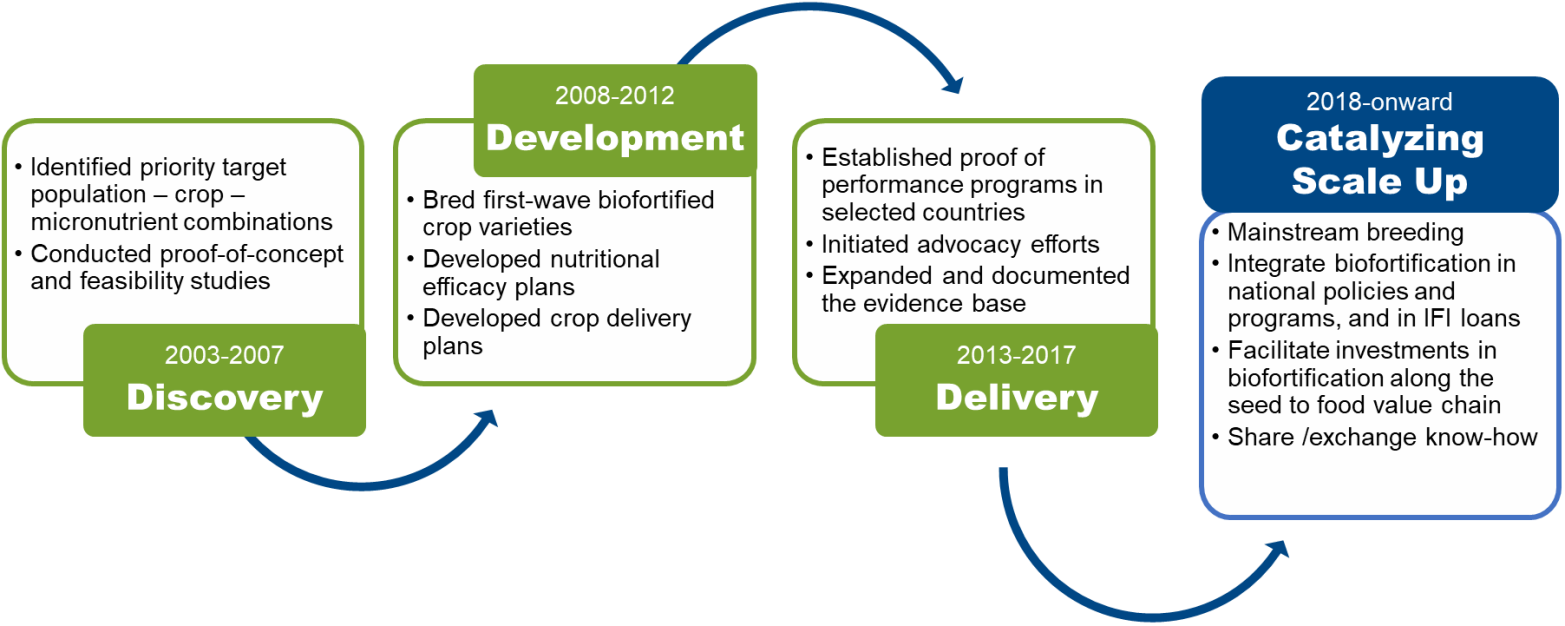
Evolution of HarvestPlus.

Research (of all disciplines) led by HarvestPlus followed the four distinct phases of the program as depicted in **Figure 1**. Even though this figure is linear, feedback loops between the phases (depending on micronutrient-crop-country context) were significant, and was informed by the ever-evolving and growing body of research, both by HarvestPlus/CGIAR and others. Socio-economics research in the discovery phase included modelling to assess the potential impact and cost-effectiveness of biofortification so as to inform the targeting of breeding investments (micronutrient-crop-country) for maximum impact potential (see e.g., Meenakshi et al., 2010). Once target combinations were selected for investment in biofortification, socio-economics research conducted in this phase also included farm household level surveys in the rural areas of targeted LMICs to shed light on to which staple crop varieties were popular at the time and why, so as to help position biofortified varieties - once released - for optimal adoption potential (see e.g., Hossain et al., 2012).

In the development phase, consumer acceptance studies were conducted with the first wave biofortified varieties to assess not only acceptability of the food made with these new varieties, but also to identify effective “nudges” or “drivers”, such as awareness campaigns, in increasing demand for biofortified food (see e.g., Oparinde and Birol, 2019). The first (cost-) effectiveness study, a randomized controlled trial measuring the effect of a program that delivers orange sweetpotato (OSP) planting material on reducing vitamin A deficiency was also conducted in this phase. The favorable results of this study (see e.g., Gilligan, 2012) led to increasing investments in the development and delivery of biofortified crops. Existing seed to food staple crop value chains were assessed in this phase to understand potential opportunities and challenges (and entry points) for delivery and eventual scaling of biofortified varieties once released (see e.g., Murekezi et al., 2013).

In the delivery phase, a program monitoring, evaluation and learning system (MEL) was developed and implemented for 20 national-level biofortification programs catalyzed by the HarvestPlus program (see e.g., Rodas-Moya et al., 2022). Also in this phase, as country programs matured, outcome and impact assessments were conducted to understand adoption and diffusion of biofortified varieties (see e.g., Asare-Marfo et al., 2016a; Asare-Marfo et al., 2016b); and using this and available program cost data, analyses were conducted on cost-effectiveness, cost-efficiency and cost-benefit of country programs and delivery models associated with each crop (see e.g., A4NH, 2019).

In the final, catalyzing scale up phase, all of the information gathered in the previous phases of the program, as well as available evidence generated by others, was stylized into strategies for scaling biofortified crops and foods within food systems. Also in this final phase, a robust knowledge management system was developed and this resulted in various knowledge products, such as the evidence brief (see link: https://www.harvestplus.org/wp-content/uploads/2021/12/Biofortification_The-Evidence.pdf) and user-friendly tools, such as a comprehensive list of national policies and regulations that include biofortification (see link: https://www.harvestplus.org/home/advancing-policy/national-biofortification-policies-and-regulations-2/). These resources were integrated into a knowledge management, mobilization and exchange platform (see link: https://www.harvestplus.org/biofortification-hub/toolbox/) whose aim is to equip public and private sector, donor community and other investors with information to encourage and enable the replacement of non-biofortified staples with their biofortified counterparts.

The next section describes materials used to develop the overall socio-economic research program and the approach we followed to develop this article. The following section discusses the socio-economic methods used in each one of the phases introduced above (**Figure 1**) and explains how the results of these studies were utilized by the program. The final section discusses the key lessons learned from conducting interdisciplinary research in general, and the role of socio-economic research in supporting plant breeding in particular.

## 2 Materials and methods

Given that in its essence HarvestPlus is a breeding program for nutrient-dense biofortified crops, one material without which the portfolio of socio-economic research presented here could not have been conducted is the biofortified varieties of crops developed by the breeders. Many socio-economic studies conducted during the development and delivery phases, such as consumer acceptance studies and effectiveness studies, and of course the MEL and impact assessments of the program in general and country programs in particular, depended on the availability of the biofortified planting material, which depended on the availability of genetic variation (of the micronutrient with which the crop was to be biofortified) in the gene banks. Biofortified varieties were bred not only to be nutrient-dense but also climate-smart, high-yielding and responding to farmers’ and consumers’ various needs and preferences whether it be resistance to a particular pest or a certain texture needed for processing into a commonly consumed food. These varieties were developed in close collaboration between the CGIAR and national agricultural research systems (NARS), and following several years of testing for the abovementioned attributes, they were released by the national governments for planting by farmers in their countries (Douthwaite, 2021; Douthwaite et al., 2022).

**Figure 2:**
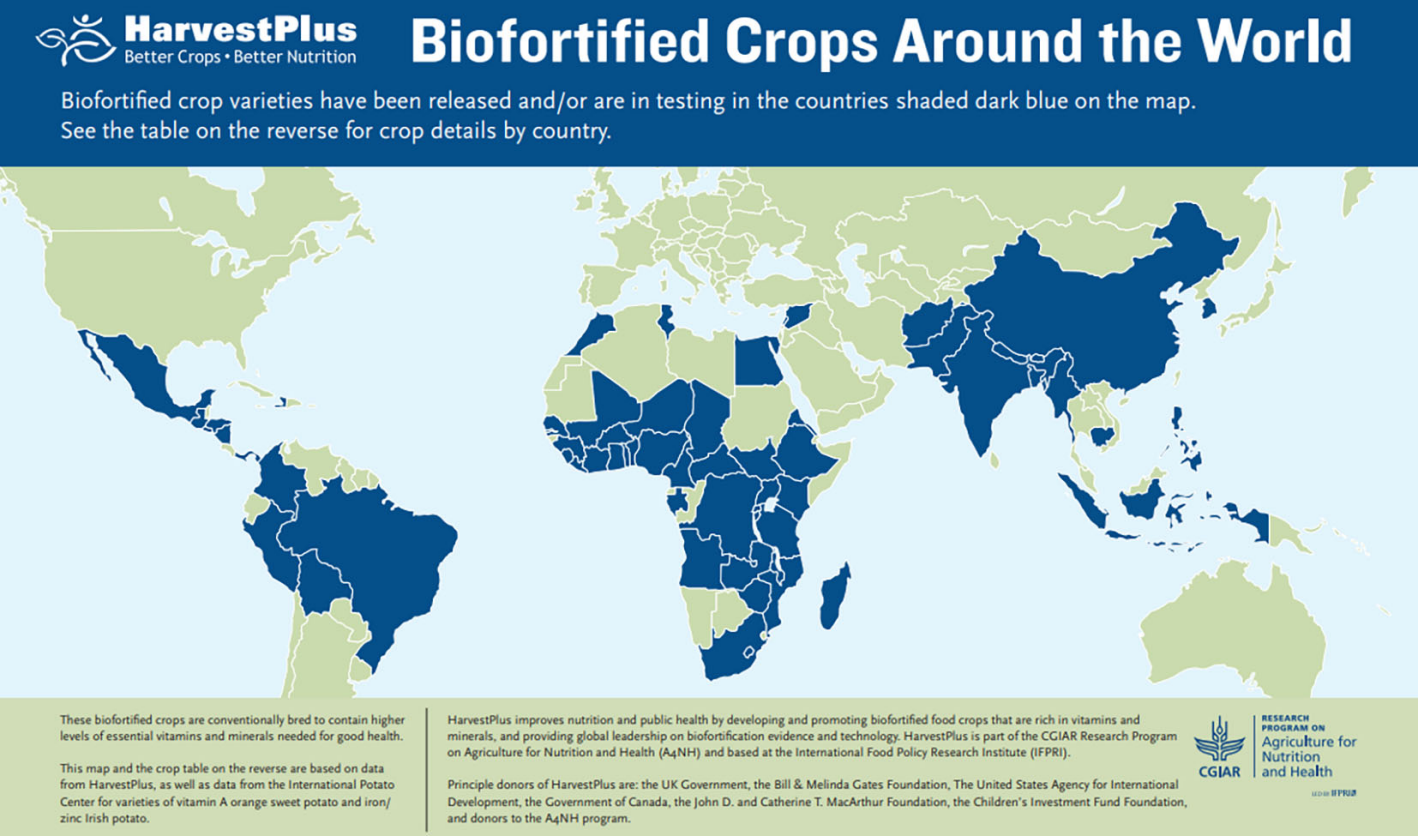
Biofortified crops around the world, blue color indicates the countries where at least one biofortified crop variety was released.

Release of biofortified varieties of crops, started as early as 2004 with Vitamin A OSP and iron beans. By the end of 2021, 422 varieties of 12 staple crops were released for planting across 41 countries with hundreds of more varieties in the release pipeline in these and additional 22 countries (**Figure 2**). These biofortified crop varieties and the number of releases thereof are: vitamin A banana/plantain (14), cassava (28), maize (71) and OSP (147); iron beans (79), cowpea (10), lentils (12) and pearl millet (11); zinc sorghum (1), maize (11), rice (16) and wheat (22). For further details of biofortified crop varieties released (including varietal names, local names, yields, and countries of release), see HarvestPlus data-base for biofortified crops released (link: https://bcr.harvestplus.org/).

To develop this paper, we have revisited the strategies and key proposals developed for each phase of the program; reviewed various donor and annual program reports, and discussed the key socio-economic research studies either led or funded by HarvestPlus with the principal investigators (PIs) who were either employed by or have collaborated with the socio-economic research component of the HarvestPlus program. Several of these PIs have reviewed and commented on the earlier drafts of this paper. We have also reviewed the full set of socio-economics documents originated from HarvestPlus program, including peer-reviewed journal articles, study reports, HarvestPlus working papers and research for action papers, discussion papers published by partner organizations (such as the International Food Policy Research Institute [IFPRI], and Global Alliance for Improved Nutrition [GAIN]), conference presentations, as well as unpublished manuscripts that are in the institutional library of HarvestPlus.

## 3 Socio-economic research methods and results

### 3.1 Evaluating the potential of biofortification interventions for impact

Two streams of research were conducted to help inform targeting of plant breeding and delivery investments for biofortification.

#### 3.1.1 Ex ante cost-effectiveness studies

The first was developed very early on in the discovery phase, before any other research or intervention, to assess the cost-effectiveness of potential biofortification interventions in improving health outcomes, and to compare cost-effectiveness of potential biofortification investments to those of alternative investments. Such *ex ante* analysis, relies on educated guesses with regard to intervention costs and possible benefits. Since there are many unknowns, typically scenarios are developed with the most conservative of these using maximum program costs and minimum benefits. If such “conservative” cost-effectiveness figures are still favorable, as per the World Bank criteria of what constitutes a cost-effective health investment (World Bank, 1993), then there is more confidence that biofortification is a sound investment.

For *ex-ante* analysis of potential biofortification programs, a methodology was developed to estimate the health benefits of biofortified staple crops stemming from increased intake of micronutrients: iron, zinc and vitamin A. These benefits are many [and nutrition research unearths many more each year – such as the recent findings on zinc and non-communicable diseases (Pompano and Boy, 2021)] and vary across micronutrients and target populations, ranging from reducing the incidence of diarrhea in children or of night-blindness in pregnant women to preventing child mortality or cognitive development impairments in children, and hence are difficult to compare. Disability-adjusted life years (DALYs) approach was adopted from public health economics literature. This approach circumvents the need to value life in monetary terms by calculating the number of years lost as a result of disease burden, by weighing adverse health outcomes caused by the disease (in this case micronutrient deficiency) according to each outcome’s severity and duration (Stein et al., 2005; Edoka and Stacey, 2020). As explained in greater detail in Bouis et al. (2020), this approach, for example, assigns preventing a few days of diarrhea getting a much lower relative weight in the calculations than preventing a child death. The overall health impact of an intervention can then be aggregated in terms of the number of DALYs saved by reduction in the duration of each one of these health outcomes, and the total estimated costs of the intervention can be divided by this total number of DALYs saved to assess cost per DALY saved, i.e., cost-effectiveness.

A DALY tool was developed for each one of the micronutrients. Realization of the benefits of biofortification has a long-time horizon (30 years) as it takes time for biofortified varieties with full micronutrient breeding target levels to be released for planting, and to replace non-biofortified varieties in farmers’ fields and consumers’ plates, while it also takes time for many of the health benefits of micronutrient deficiency reduction to surface among the consuming populations. For several potential micronutrient-crop-country combinations of biofortification interventions, pessimistic (i.e., conservative) and optimistic scenarios were simulated with assumptions about future coverage (adoption and consumption), costs (of breeding and delivery) and micronutrient content the biofortified crop can deliver. This tool measures the reduction in prevalence of micronutrient deficiency among target populations as a result of biofortification programs, and calculates the cost-effectiveness (i.e., cost per DALY saved) of the biofortification intervention, thereby allowing comparison thereof with the cost per DALY saved for other micronutrient interventions, such as fortification and supplementation.

**Figure 3:**
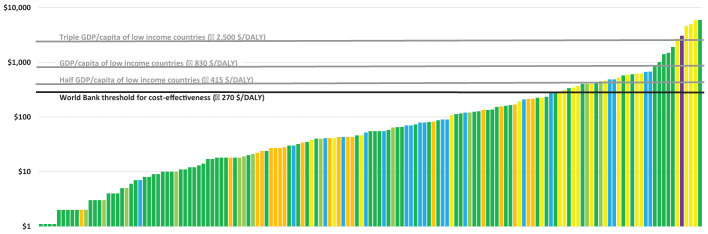
Cost-effectiveness of micronutrient deficiency reduction interventions.

Ex ante analyses conducted for several micronutrient-crop-country combinations (see e.g., Meenakshi et al., 2010; Birol et al., 2014), as well as a review of such analyses (Lividini et al., 2018) found that for most cases biofortification is highly cost-effective according to the World Bank criteria (World Bank, 1993). Analysis conducted by the Copenhagen Consensus based on early estimates of Meenakshi et al. (2010) converted the health benefits into monetary values and found that every USD invested in biofortification resulted in USD 17 of benefits (Horton et al., 2008); and ranked interventions that reduce micronutrient deficiencies, including biofortification, among the highest value-for-money investments for economic development. Comparison of cost-effectiveness of biofortification with those of other interventions targeting micronutrient deficiencies, such as supplementation and fortification (Meenakshi et al., 2010; Birol et al., 2014; Bouis et al., 2020) revealed biofortification to be more cost-effective than these other interventions in most cases, and exceptions typically involved scenarios with low substitution and/or consumption of the staple crop (Lividini et al., 2018). **Figure 3** below reproduced from Bouis et al. (2020) ranks cost-effectiveness results from various analyses of micronutrient interventions and finds biofortification interventions to be highly cost-effective in many cases (i.e., for many micronutrient-crop-country combinations).

More-in-depth studies modelling the micronutrient program portfolios looking at biofortification, fortification, and supplementation, such as for iron in India (Rajasthan) (Fiedler and Lividini, 2015), vitamin A in Zambia (Fiedler and Lividini, 2014a) and zinc in Bangladesh (Fiedler and Lividini, 2014b), all found biofortification of the key staples to be one of the most cost-effective strategies for tackling the deficiency of the micronutrient which can be addressed through biofortification. For a review of other studies comparing micronutrient interventions – especially fortification and biofortification- as well as how these interventions can complement each other, see Mkambula et al. (2022).

Overall, *ex ante* analysis results have not only helped HarvestPlus raise funding for biofortification, but also informed prioritization of investments. For example, following the first *ex ante* analysis presented in Meenakshi et al. (2010) which revealed that countries in Asia and Africa could benefit the most from biofortification, especially for mineral and vitamin A biofortification of crops, respectively, compared to the Latin America and the Caribbean (LAC) region, HarvestPlus focused its investments and efforts in South Asia and Sub-Saharan Africa regions.

#### 3.1.2 Biofortification prioritization index and tool

**Figure 4:**
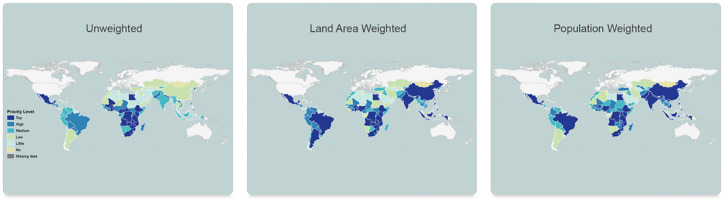
BPI for vitamin A maize, unweighted, land area weighted and population weighted.

The second research stream aimed at developing a crop specific prioritization index ranking LMICs according to their impact potential. Developed in 2013 and called the biofortification priority index (BPI), this index uses country-level data and for each staple crop it calculates the geometric mean of three sub-indices: production and consumption indices for biofortifiable staples and a micronutrient deficiency index for the micronutrient with which the crop can be biofortified (Asare-Marfo et al., 2013). **Figure 4** below shows the BPI for vitamin A maize, for which the countries are ranked from top priority to low and no priority, with majority of the top and high impact potential countries being in Sub-Saharan Africa.

Following the positive feedback received from breeders and donors regarding the usefulness of the index, a user-friendly and open access tool was developed (BPI - HarvestPlus https://bpi.harvestplus.org/). The BPI tool was first published in 2015 based on the 2013 analysis, and the tool was updated in 2019 with most recent data and information and to include additional crops (see Herrington et al., 2019) and features such as links to the varietal releases and evidence on outcomes nutrition both of which get updated annually.

Based on the feedback received from various users, the BPI tool is known to be very helpful in informing investment decisions pertaining to biofortification. For example, crop breeders especially in the CGIAR have been known to look at the crop specific BPI maps to identify for which agroecological zones/countries they should breed biofortified varieties for the highest potential impact (e.g., Steve Beebe, CIAT bean breeder; Elizabeth Parkes and Peter Kulakow IITA cassava breeders, personal communication, 2018-2019).

Similarly, agriculture, nutrition and health departments of international financial institutions, such as the World Bank’s agricultural practice for Sub-Saharan Africa, have used the BPI to identify in which countries in the region an investment in a certain biofortified crop could yield in the highest impact (e.g., Mark Cackler, Manager Agriculture and Food Security, World Bank, personal communication 2019). At the country level, national decision makers, for example ministry of agriculture considering including biofortification programs in their breeding programs at their national agricultural research systems, or ministries of health and education thinking about introducing biofortified crops in school feeding programs, can visit the country pages in the BPI tool to see which biofortified crops are potentially most impactful at scale in their country and the availability of these crops (personal communication from various country level presentations of the tool).

Humanitarian agencies can also use the BPI, for example World Food Programme (WFP) policy emphasizes procurement of biofortified staples, when possible, to improve not just food but also nutrition security. The population weighted version of the BPI, could help WFP understand in which countries biofortification would have the biggest population-level impact, especially for most vulnerable populations, namely women and children, which the population-weighted BPI focuses on. Similarly, the BPI was also used by various international NGOs, such as World Vision, when making decisions on where to introduce biofortified crops in their national rural programming (Brian Hilton, Food Security Advisor, World Vision, personal communication, 2016). Finally private companies, many of which are increasingly committing to sustainability goals, such as by taking the Nutrition for Growth Responsible Business Pledge, could want to invest in R&D and scaling of seeds of biofortified varieties. Given the seed sales for a certain biofortifiable crop would be higher in countries where larger areas of production are allocated to that crop, land-weighted BPI would reveal the biggest markets for the biofortified crop. Private seed companies have also been known to use the BPI to plan their investments (Ashishs Wele, Nirmal Seeds, India, personal communication 2016).

### 3.2 Assisting the design of micronutrient- crop-country specific biofortification programs

Following identification of the micronutrient-crop-country combinations to invest in; development and release of biofortified varieties of crops started to take off during the first two phases of the program. Given the imminency of the design and implementation of the country delivery programs, the socio-economics team conducted three streams of research for each program micronutrient-crop-country combination that was targeted.

#### 3.2.1 Varietal adoption studies

The first research stream, focused on the farmers, i.e., the future supply side of biofortified crops, to understand their current choices, and opportunities and challenges they face that could hinder or encourage adoption of biofortified varieties. Collectively called varietal adoption studies, these studies collected - where possible/applicable - nationally, or state level, representative data from smallholder farmers who grow the crop of focus.

The main information collected through these studies were the names, planting material source, and agronomic (as well as processing and consumption) characteristics of the [crop] varieties these farmers cultivated at the time. Data on input and output market access, farm and household level characteristics (e.g., farm size, household income and assets, household access to subsidies; who in the household is the main decision-maker when it comes to making varietal decisions and their gender, age and education), area planted to the crop, farmers’ planting material sources (formal vs informal; public or private) and costs of acquiring planting material, as well as farmers’ trusted sources of information for new varieties were also collected. Since the aim of biofortification was to improve the micronutrient intake of rural smallholders who consume what they produce, data were collected on the use of crop output (consume vs sell, livestock feed, save as seed); information on who in the household makes the cooking/feeding/nutrition decisions; food preparations the crop is consumed as, and households’ current dietary diversity.

From 2004 to 2013, varietal adoption studies were conducted for wheat in India and Pakistan, rice in Bangladesh and India, pearl millet in India, maize in Zambia, sweet potato in Uganda and beans in Rwanda and Uganda. The main challenge of these studies was the identification of varieties as improved vs traditional, and required close collaboration between the socio-economics team and the CGIAR and NARS breeders. This challenge was especially hard for non-hybrid crops whose seed are not regularly (if at all) purchased in formal seed systems and whose varieties don’t have significant morphological differences across improved vs. traditional types.

Data collected through this research stream not only unearthed that for several crop-country combinations, adoption of modern, i.e., improved varieties developed by the CGIAR and NARS was not as widespread as thought, but also highlighted what kind of varietal attributes smallholder farmers were looking for, and where they were acquiring their planting material and information thereon. Further econometric analysis conducted with the data from these studies helped identify factors (farmer, market, agro-ecological, policy level) that encouraged or hindered adoption of modern varieties. Some of the published examples of the analysis conducted include Hossain et al. (2012) for rice in Bangladesh and eastern India, Nazli and Smale (2016) and Battese et al. (2017) for wheat in Pakistan, Birol et al. (2015a) and Smale et al. (2016) for pearl millet in India, and Smale et al (Smale et al., 2014; Smale et al., 2015a). for maize in Zambia. There are also several HarvestPlus working papers, project reports and data sets available online (see e.g., Smale and Mason, 2012; De Groote et al., 2014; Tahirou et al., 2015; Oparinde et al., 2016b; Larochelle et al., 2018).

**Figure 5:**
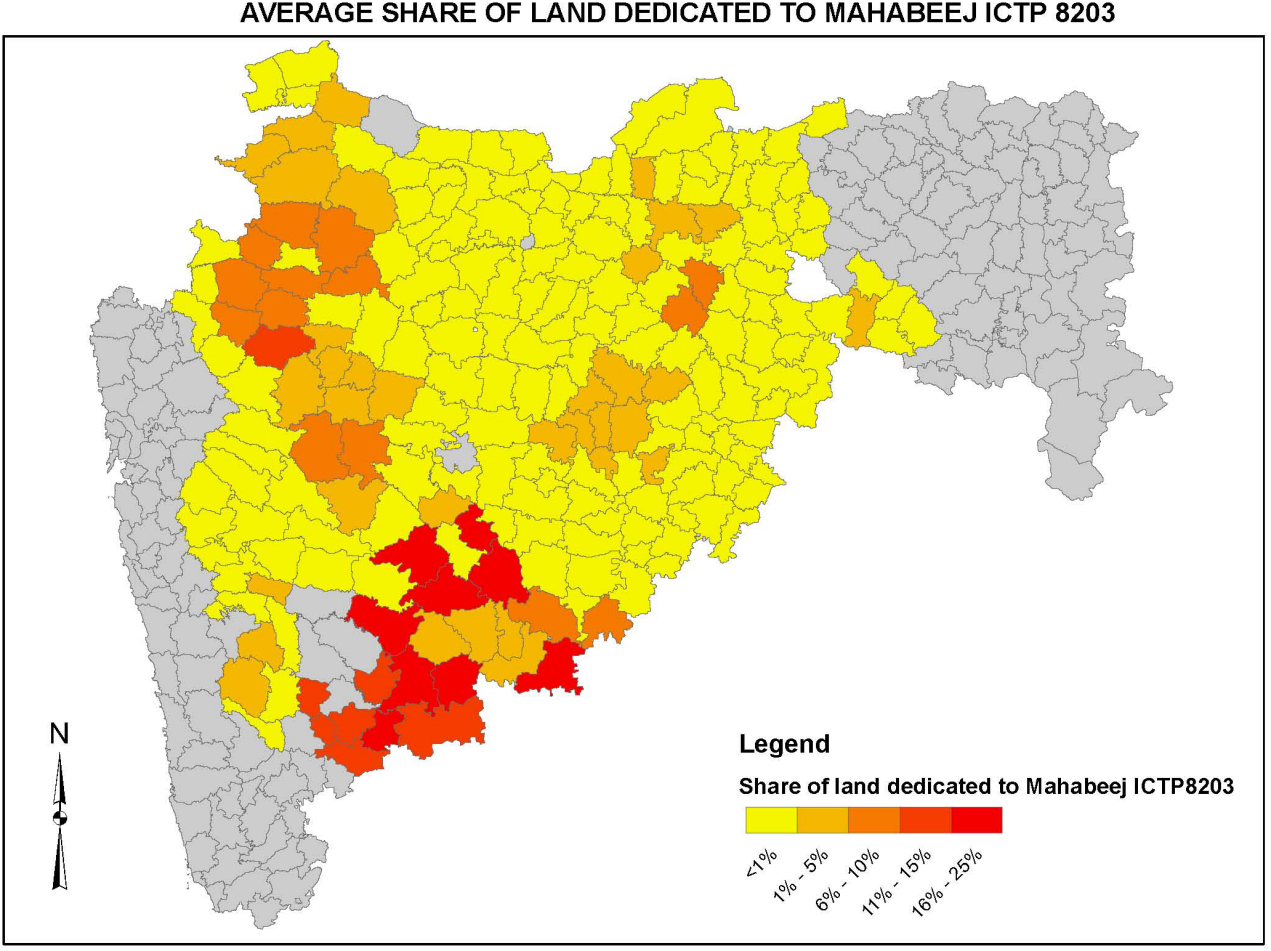
Varietal adoption map for ICTP 8203, Maharashtra, 2011.

Information on which varieties farmers are currently growing and which varietal attributes they appreciate was fed back to the breeding teams at the CGIAR and NARS. Information collected on the factors that affect adoption, and farmers’ [trusted] sources of information and planting material was used in the development of micronutrient-crop-country level biofortification delivery programs. Data on the adoption of improved varieties was also used to develop varietal maps which were useful for both plant breeders and delivery programs. **Figure 5** below, for example, shows the adoption of an improved open pollinated pearl millet variety, namely ICTP 8203 in 2011 in the Maharashtra state of India. An iron-biofortified version of this variety, ICTP 8203-Fe which was later called *Dhanshakti -* was released for production in 2012, and the subsequent efforts to promote and deliver *Dhanshakti* was focused on the darker red and orange areas in the figure below. Finally, the information generated through this research stream was also useful for other research initiatives, such as the Diffusion and Impact of Improved Varieties in Africa (DIIVA) project, which collaborated with HarvestPlus socio-economics team both during development of the overall methodology and implementation of the data collection efforts in several countries (Walker and Alwang, 2015).

#### 3.2.2 Consumer acceptance studies

For biofortification to be successful in its aim of alleviating micronutrient deficiencies, adoption of biofortified varieties by farmers is necessary but not sufficient. Consumers of the food made with these crops should also accept, in fact prefer, these varieties to their non-biofortified counterparts. Consumer acceptance studies were conducted from 2008 to 2018, as biofortified varieties became available. These studies used field experiment methods and sensory evaluations to assess consumers’ valuation (in terms of their willingness to pay) and evaluation (of various organoleptic characteristics) of foods made with biofortified varieties of crops vis-à-vis food made with the most commonly consumed variety at the time.

There have been various consumer acceptance studies for several biofortified crops (iron pearl millet and beans, zinc rice, vitamin A cassava, maize and orange sweet potato) conducted by both HarvestPlus and others, as well as critical reviews of these studies (see e.g., Birol et al., 2015b and Oparinde and Birol, 2019). Earlier consumer acceptance studies focused on rural consumers (as producer-consumers of staples are the priority for the impact of biofortification). However, after varietal adoption and value chain research revealed that urban demand pull could act as a catalyzer for rural adoption, and that smallholder farmers value those varieties that have buyers at the market (even if farmers’ first and foremost use of the harvest is for household consumption), consumer acceptance studies with urban consumers were also conducted (e.g., see Murekezi et al., 2017; Oparinde et al., 2017).

Consumer acceptance studies were especially important for vitamin A biofortified crops which change color (as well as taste and to some extent texture) due to increased beta carotene content that comes with vitamin A biofortification. Consumer acceptance studies were conducted for vitamin A maize – which tends to be dark yellow to orange as opposed to the commonly consumed white – in Zambia (Meenakshi et al., 2012); vitamin A orange sweet potato – which tends to turn a deep orange color as opposed to commonly consumed cream color – in Uganda (Chowdhury et al., 2011); and vitamin A cassava - which is light to deep yellow as opposed to white/cream varieties – commonly consumed in Nigeria (Oparinde et al., 2016a). All of these studies have revealed that color change was not an impediment to consumers being willing to purchase and consume biofortified varieties – in many cases even in the absence of information about the nutrition and benefits of vitamin A varieties. Consumers were willing to pay as much – if not more – for food made with biofortified varieties of crops; they liked the taste of these varieties as much if not more than food made with non-biofortified varieties. This information was indeed validating for the potential impact of biofortification at scale. It has also been useful for the breeders to ensure that next generations of biofortified varieties developed have all the attributes consumers are looking for (such as improved dry matter content for vitamin A orange sweet potato and cassava).

This body of research also investigated “nudges” to facilitate the acceptance of food made with biofortified varieties of crops. Almost all studies looked at the role of information or awareness raising with regards to biofortification and its potential nutrition and health impacts. Overall having information about the health benefits of biofortification meant higher acceptance and higher willingness to pay, i.e., higher valuation which means higher economic benefits from consuming biofortified foods. In order to inform promotion and awareness raising efforts, these studies looked at use of type of media (e.g., radio messaging vs. community leader messaging as in Meenakshi et al., 2012); source of information (e.g., celebrity vs public officer as in Oparinde et al., 2016a or through social networks, i.e, consumer to consumer as in Muange and Oparinde, 2018); frequency of information (Pérez et al., 2018, Oparinde et al); information framing as loss/negative (lack of micronutrients make you sick) vs. gain/positive (micronutrients in diet make you healthy) (see e.g., Oparinde et al., 2016a); amount of information (e.g., Oparinde et al., 2017).

For biofortified crops that do not have any visible traits (i.e., zinc and iron in biofortified crops does not change the color or taste) studies tested the role of processing (e.g., Herrington et al., 2022), as well as certification and branding on engendering demand, and the source of certification/branding (international vs local) on consumer acceptance (see e.g., Banerji et al., 2016). Overall, these studies both informed delivery programs promotion and marketing activities and helped push the frontier of the revealed preference - field experiment research. For example, these studies compared different preference elicitation methods (e.g., Banerji et al., 2018a); asked participants to pay out of pocket in experiments to test if windfall income bias (common with such field studies) can be minimized without compromising study participation (see e.g., Oparinde et al., 2016b); and estimated consumers’ willingness to pay to replace non-biofortified food with biofortified food (Banerji and Rampal, 2020).

#### 3.2.3 Value chain studies

The third stream of research under this category included assessment of seed to food value chains for biofortifiable staple crops to understand the potential bottlenecks and opportunities; winners and losers, as well as enabling mechanisms to facilitate the scaling of biofortification. Since the target staple crop-country combinations were very well studied by both implementation and academic research, a majority of this work could be conducted through literature and document reviews, and consulting with key value chain experts in CGIAR centers as well as key value chain actors in the program countries.

Research questions asked included those to understand how value chains might work (or not) for biofortified varieties – the flow from seed to grain to food – while minimizing losses of their nutritional value; if or what kind of mechanisms could be introduced/tweaked for biofortified products (e.g., branding, certification, technology for testing for micronutrient content, financing, technology for linking value chain actors) and where in the value chain would it make most sense to introduce these mechanisms.

These studies varied from qualitative value chain studies of informal seed systems, such as those for beans in Rwanda (Murekezi et al., 2013) and cassava in Nigeria (McNulty and Oparinde, 2015), and profit assessment of small- and medium-scale food enterprises that produce food made with biofortified varieties (Bamire et al., 2018), to comprehensive analysis of the formal seed industry for example for hybrid maize in Zambia (Smale et al., 2015b), and hybrid pearl millet in India (Karandikar et al., 2018), as well as informal seed networks (see e.g. Oparinde et al., 2016b).

The findings of these studies were instrumental in the development of the delivery models. For example contract farming model suggested by McNulty and Oparinde (2015) for both production of biofortified cassava planting material for delivery to farmers, and biofortified cassava roots for processing, was implemented not only in Nigeria but in several of the country programs. This study’s highlighting of the importance of finance for small-scale producers led to inclusion of an innovative finance component in the scaling phase of the program. Another example is Smale et al. (2015b) which looked at the hybrid maize seed industry in Zambia and suggested that risk-sharing agreements with any seed company that takes up vitamin A maize would be essential – along with building and maintaining a unique value proposition for vitamin A maize – to facilitate scaling. Consequently, the Zambia biofortification program invested in significant awareness raising for vitamin A maize, while supporting (both technically and financially, at least at first) private seed companies’ involvement in vitamin A maize seed production.

All of these studies had a gender and inclusivity lens to understand who are the actors currently benefiting or losing in the staple crop value chains, and how/if substitution of biofortified varieties with their non-biofortified counterparts can change this dynamic in favor of more vulnerable actors (such as women traders and women owned SMEs) and to what extent (see e.g., Asare-Marfo et al., 2019). Information generated through these studies informed engagement with both the supply side (e.g., private seed companies, as well as community-based seed multipliers and budding seed sector in many countries) as well as the demand side (traders, processors and food companies of all sizes).

### 3.3 Monitoring, evaluation and learning system for country programs

As delivery programs for first waves of released biofortified planting material kicked off in several countries in/around 2011, the need for a rigorous monitoring, evaluation and learning (MEL) system arose, not only to monitor (and report on) day to day progress of implementation, but also to assess the outputs, outcomes and eventually impact generated by program implementation.

The socio-economics team quickly realized the need for a MEL system and capacity, and in 2013 put together a global MEL unit consisting of MEL specialists at country and global levels. The MEL team worked very closely with the rest of the socio-economics team, as well as with the country programs to develop crop-country specific program theories of change (ToC); identify key indicators; design methods and metrics for their measurement, and to develop the tools for collecting data. An objective of the MEL system was to be as easy to use/implement and as transparent as possible. Since in several countries delivery and hence data collection were done by partner organizations, the MEL system included a data quality assurance component as well as a training/capacity strengthening component for partners.

**Figure 6:**
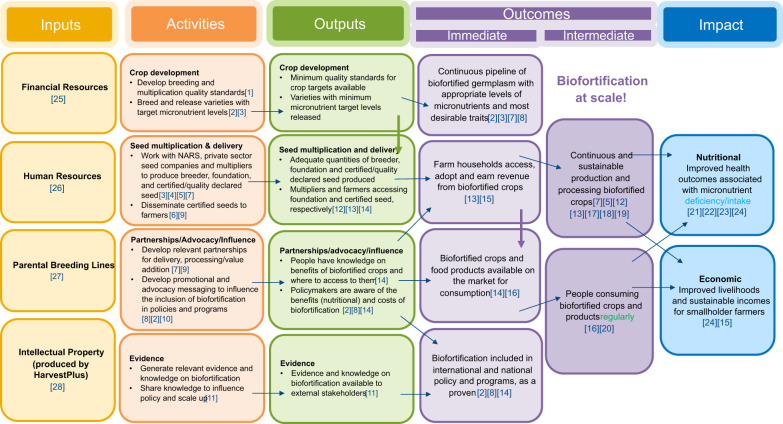
Biofortification Theory of Change. Assumptions:[1] Evidence on appropriate breeding targets developed for all crops from nutrition studies; and quality control standards (for detection of nutrient levels) and/or branding guidelines developed. [2] Necessary partnerships built with national governments and NARS and MoUs signed with CG centers. [3] Agronomic and other varietal trait requirements are met or exceeded. [4] Licensing of released varieties to private seed companies/multipliers. [5] Quality control standards are implemented. [6] Appropriate (country-specific) dissemination strategies in place. [7] Necessary partnerships built with private companies and multipliers. [8] Necessary partnerships built with key international bodies (e.g. World Bank). [9] Develop criteria for working with private sector companies and evaluate prioritized targets. [10] Guidelines on biofortification developed as a result of high level stakeholder meetings with FAO and WHO. [11] Knowledge management tools, processes and outputs developed for public and private stakeholders. [12] Availability and sufficiency of biofortified planting material. [13] Farmers know where to obtain biofortified planting material. [14] Messaging is clear, accurate and targeted. [15] Farmers sell some of their harvest in the local markets. [16] Consumers are convinced of the nutritional benefits of the biofortified crop. In addition, biofortified foods have the same or better taste and cooking quality. [17] Farmers are willing to increase land cultivated to biofortified varieties (by replacing biofortified seed with regular crop seed area or opening new farm land). [18] Farmers spread information about biofortified seeds and crops and share planting material and output/harvest. [19] Full government support through programs and policies in favor of biofortified crop varieties. [20] Caretakers in target households have accepted the nutritional benefits and have access to biofortified foods and are willing to feed their household. Children are willing to eat biofortified foods. [21] Micronutrients are preserved during cooking and storage/Households use recommended storage and preparation methods that do not result in high nutrient loss for biofortified crops. [22] Consumption of biofortified crops working together with other nutrition interventions (e.g. fortification, dietary diversity)/Biofortified crops. [23] Biofortified foods are eaten in sufficient quantities on a regular basis. [24] There are no other underlying health conditions. [25] Fundraising efforts yield additional financial resources. [26] Human capital and the right skills are in place to do the various types of work. [27] CG centers produce parental breeding lines. [28] Evidence on biofortification effectiveness/efficacy and sufficient nutrient target levels for biofortified crops is available.

The MEL system was developed to include 30 indicators: 5 process indicators (e.g., numbers of information events, people attending these events, publications), 10 output indicators (e.g., quantity of biofortified planting material produced and delivered to farmers, number of people trained and biofortified varieties released), 12 outcome indicators, and 3 impact indicators. Data on the process and output indicators were collected regularly through made for purpose and easy-to-use forms and tools (such as attendance registers to track the numbers of people trained, or planting material distribution forms signed by recipients of biofortified planting materials. Regularly collected and reported data enabled both country and global teams to better understand the implementation progress, ensure implementation fidelity, identify red flags and account for resources used. **Figure 6** below shows the generic ToC for biofortification programs, and **Table 1** presents the full list of indicators collected and estimated.

**Table 1:** List of indicators.

Type	Indicator
Process	1. No. of tools developed 2. No. of information sharing events 3. No. of people attending information sharing events* 4. No. of Information, Education and Communication (IEC) materials developed 5. No. of publications produced
Output	1. Quantity of planting material produced that is available for next planting season 2. Quantity of planting material delivered to farmers 3. No. of households reached through delivery of planting material for production purposes* 4. Quantity of inputs distributed by HarvestPlus and its contracted partners 5. No. of varieties under on-station research 6. No. of varieties under field testing 7. No. of varieties released 8. Quantity of early generation seed produced 9. No. of people trained * 10. No. of partners HarvestPlus is working with
Outcome	1. No. of households (HH) reached with planting material for production through farmer to farmer 2. No. of HH reached through acquisition of planting material from the ‘seed market’ 3. No. of HH reached through acquisition of the biofortified food products for consumption 4. % of farmers who planted biofortified crops after acquisition 5. % of area planted with biofortified crops 6. Area planted with biofortified crops by farmers 7. % market share of biofortified foods 8. Quantity of biofortified crops harvested/produced 9. Proportion of biofortified crops or their products that is consumed by the farming HH 10. % of women of reproductive age who consume biofortified crop products 11. No. of children under five who are eating biofortified crop products 12. No. of public sector policy/plan/guidelines/strategy, documents developed
Impact	1. % of the estimated average requirement (EAR) delivered disaggregated by crop and target demographic group 2. Change in the prevalence of inadequate intake of target micronutrient in project intervention areas disaggregated by geographic location 3. Number of DALYs averted

* disaggregated by sex.

Data on outcome indicators were collected through adoption surveys and effectiveness studies (explained in greater detail below), and farmer feedback and outcome monitoring surveys. Farmer feedback studies (akin to customer feedback studies conducted in high income countries) were implemented among recipients of the planting materials of the first wave varieties to understand farmers’ experiences with the varieties and the delivery model employed. This “dip-stick” type surveys enabled early feedback to plant breeders and implementation programs on what was going well and what could be improved upon. Outcome monitoring surveys are conducted in each one of the program countries every two to three years in sentinel sites selected purposefully to represent program implementation intensity and/or delivery model used. These surveys are conducted on a representative sample of households and aim to capture not only adoption (and sources of planting material) of biofortified varieties but also area planted, quantity harvested, quantity purchased from markets (if any) and whether or not women and children in the households consumed food made with biofortified crops.

These surveys are complemented by qualitative studies (key informant interviews and focus group discussions) to understand [gendered] decisions behind adoption, sales and consumption; intrahousehold allocation of biofortified foods, and other reflections/feedback on the delivery models implemented and performance of varieties themselves. These qualitative studies also helped develop lessons learned (learning component of MEL) and together with the survey results facilitated adaptive programming. Examples of outcome monitoring surveys include HarvestPlus (2017, HarvestPlus, 2018a; HarvestPlus, 2018b; HarvestPlus, 2018c) for Rwanda, Zambia, Nigeria and India, respectively.

**Figure 7:**
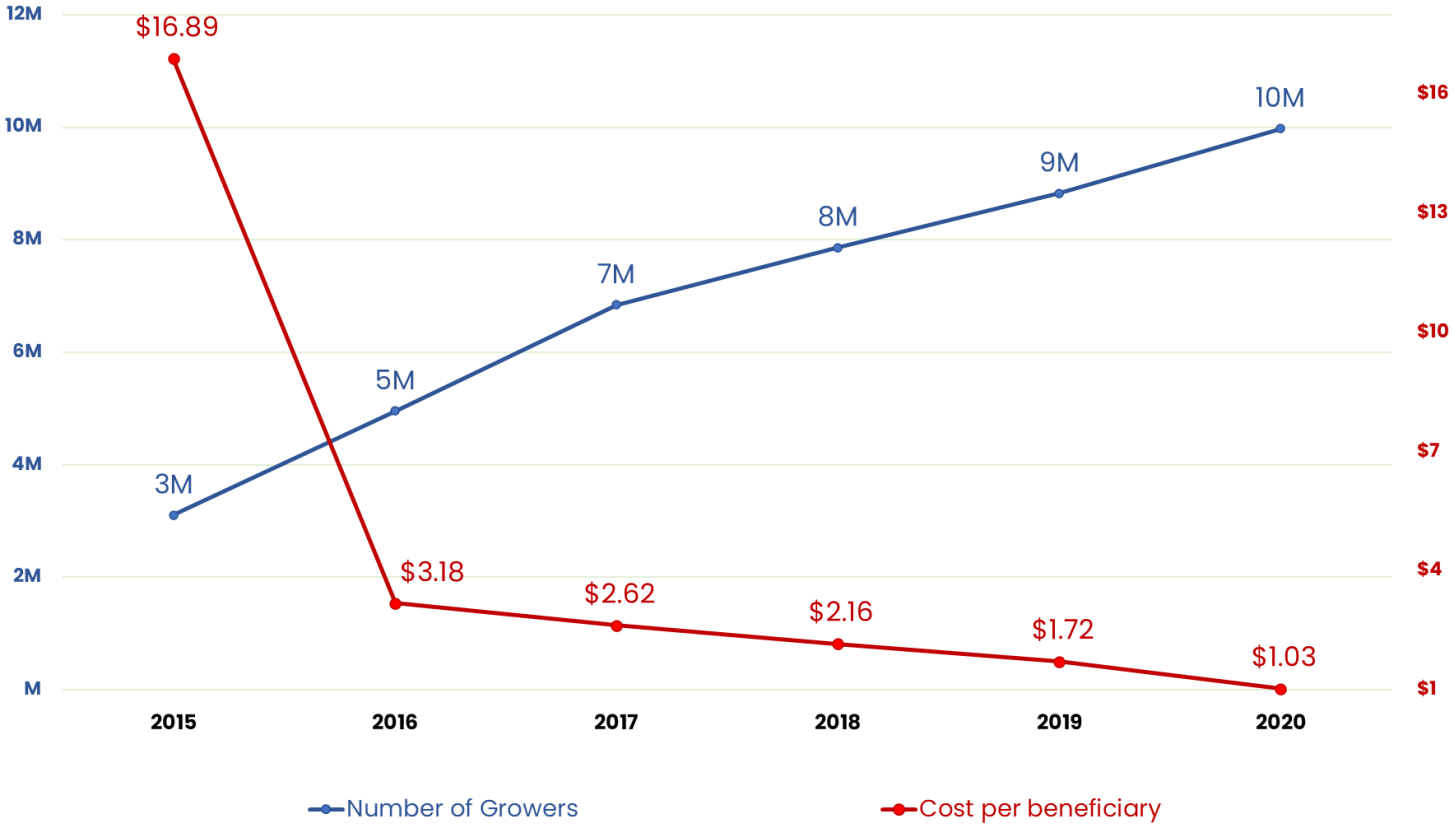
Number of households growing biofortified crops (left) vs the cost/household (right), 2012-20.

The main metric that is often reported to the donors and other stakeholders is the annual households growing numbers which are estimated by the Global Households Reached Projection Model (GHRPM). This metric shows the total number of households growing biofortified crops in any given year (Lividini, 2020). This model uses the output indicators on number of households reached with planting material, through various delivery models, as well as outcome parameters (such as adoption, disadoption, diffusion) from monitoring surveys, adoption surveys (explained below), qualitative and other studies to estimate a net, cumulative number for every year of program implementation. **Figure 7** below, computed for delivery programs across 20 countries, shows that adoption is increasing over the period 2012-2020 while the annual program cost per participant (i.e., growing household) is declining over the same time period. Number of growing households is however expected to be an under estimate of number of total benefiting households, since with increasing production, an increasing number of households are expected to purchase biofortified food from the market.

Where possible, and as per advice and guidance from CGIAR gender specialists, the indicators of this MEL system were sex disaggregated, and data collection methods included several project level Women’s Empowerment in Agriculture Index (pro-WEAI) questions (Malapit et al., 2019). MEL system was developed not just for programmatic needs but also to report to various donors whose reporting requirements and needs were varied. Therefore, development of the MEL system was very iterative and collaborative, presented on several occasions to the program leadership team, country program managers, key delivery partners and key donors of the HarvestPlus program (e.g, USAID, FCDO, BMGF) to get their feedback as well as buy-in in program implementation.

During the catalyzation phase, under HarvestPlus socio-economics team’s leadership a collective was formed to include experts from Global Alliance on Improved Nutrition (GAIN), International Potato Center (CIP) and Wageningen University of Research (WUR), with funding as well as technical guidance from the CGIAR Agriculture for Nutrition and Health (A4NH) and European Commission’s Food Fortification Advisory Services (2FAS). Based on GAIN’s experience on MEL of large-scale food fortification programs; CIP’s experience with delivery of orange sweet potato and the MEL activities thereof, and WUR’s MEL expertise of nutrition programs, the collective developed a MEL system for large scale biofortification activities. The hope of the collective is that the key indicators will be collected by national governments, international agricultural and health/nutrition data collection efforts (e.g., FAOSTAT, DHS), private sector and related indices (such as the Access to Nutrition Index) so that national level scaling and hence global biofortification efforts can be tracked. The details of this system can be found in Rodas-Moya et al. (2022) and Biofortification MEL Collective (2022).

### 3.4 Estimating impact

In addition to the GHRPM and forecasting and tracking model described above, rigorous impact evaluations (also known as effectiveness studies) and impact assessments were also conducted. These studies not only helped build the robust evidence base, but also provided valuable input to the tracking and forecasting models.

#### 3.4.1 Biofortified variety adoption studies

Once substantial biofortified planting material delivery activities have taken place, biofortified variety adoption studies (also called impact assessment studies) are conducted. These studies – just like the varietal adoption studies conducted in the first two phases – are designed to be nationally representative of the small-scale producers that are growing the crop of focus in the country. The main aim of these studies is to assess the (depth and breadth of) adoption of biofortified varieties. Data are collected to assess the source of biofortified planting material (e.g., from the program, market purchase, farmer-to-farmer diffusion), area planted to biofortified varieties and output; use of biofortified harvest (home consumption, sale, save as planting material); factors (farm, household, market, policy, and delivery model related) hindering or facilitating adoption of biofortified varieties; farming households’ feedback on the delivery models used and the varieties themselves.

An adoption study was conducted on a nationally representative sample of rural bean farming households in Rwanda. The study found that after four years of iron bean delivery efforts, 28 percent of households had planted at least one iron bean variety in at least one of the past eight seasons, and in 2015 iron beans made up almost 12 percent of national bean production with 80 percent of iron beans produced being consumed on-farm. The study also found high awareness of iron beans (67% of bean farmer had heard about iron beans), significant farmer to farmer diffusion rates (with 40% of adopters getting the iron bean from their social networks) and adopting households allocating increasing proportions of bean area to iron beans (from 48 percent in season one to 70 percent in season six) (Asare-Marfo et al., 2016a; Birol Vaiknoras et al., 2019). Further analysis of this data showed that adoption of the most popular iron bush bean variety in Rwanda resulted in a yield gain of 20-49 percent over traditional bush bean varieties. This effectively increased the length of time beans are consumed from own production (as opposed to through purchased beans) by almost three weeks, while increasing the probability of selling beans by 12% (Birol Vaiknoras and Larochelle, 2021). These results indicate that iron bean production has positively and significantly improved both food and nutrition security, as well as livelihood security, among adopting households. Since 2019, iron bean biofortification program was handed over to the national agencies, who are now taking this program from strength to strength (Mudyahoto et al., 2021).

In addition to the Rwanda iron bean adoption study, one other nationally representative adoption study, namely zinc rice adoption in Bangladesh, was conducted in 2018 (Bashar et al., 2019). This study showed significant awareness of zinc rice in the country, as well as spread of the zinc rice varieties across the country and appreciation of these varieties among adopters. The intensity of adoption was however low, owing to the lack of seed availability. Given the high costs of these national level surveys, HarvestPlus socio-economics team, in close collaboration with the CGIAR Standing Panel on Impact Assessment (SPIA), advocated to have several forthcoming national agricultural surveys and living standards measurement study surveys include biofortified crops and food (as a sub-category of key crops produced and food consumed). To date biofortified crops have been included in national level surveys in Rwanda, Uganda and Zimbabwe, with many other countries in the pipeline.

#### 3.4.2 Impact evaluation – effectiveness studies

The aim of nutritional bioefficacy studies led by the nutrition team at HarvestPlus is to establish the relationship between consumption of biofortified foods and changes in micronutrient status and health outcomes in “ideal,” that is controlled, clinical settings. Conducted with target populations recruited in highly delimited settings (e.g., boarding schools, student housing, close-knit communities with well-defined boundaries), the point of intervention in bioefficacy studies is the food consumed, with a treatment group consuming the biofortified food, and a control group consuming the same non-biofortified food. By now, there is robust evidence of the efficacy of iron and vitamin A biofortified crops in reducing iron and vitamin A deficiencies, and improving associated health outcomes (see Birol et al., 2021 for a summary of this evidence). Significant progress has also been made in identifying potential biomarkers for assessment of effectiveness of zinc biofortified crops in reducing zinc deficiency (Knez et al., 2022).

An effectiveness study can be conducted once the bioefficacy of a biofortified food is ascertained, and biofortified varieties with target levels of micronutrients are released for production by farmers in the country. Effectiveness studies - which also use randomized controlled trials - are considered as the gold standard for assessing the proof of concept of biofortification as an efficacious and cost-effective population level intervention for improving micronutrient deficiency status. Implemented under “real-world” conditions, effectiveness studies are designed to measure the causal impact of interventions delivering planting material of biofortified crops on various outcomes, including farm household adoption, diffusion and consumption of biofortified crops, as well as measuring changes in relevant micronutrient intake and biomarkers to assess the impact, i.e., improvement in micronutrient deficiency status. In addition to measuring the impact of biofortification interventions on these outcomes, effectiveness studies also gather delivery cost data and investigate the cost-effectiveness of the delivery models implemented in order to make recommendations for scaling up those biofortification delivery interventions that are found to be most effective in improving micronutrient deficiency status at least cost.

To date only two effectiveness studies were published. These were implemented in Uganda and Mozambique to investigate the effectiveness of two delivery models (one more intensive and hence more expensive than the other) for disseminating planting material for a vitamin A OSP, for which full target (if not higher) varieties were available very early on in the program. As with the efficacy studies, this randomized controlled trial had treatment and control groups. In this real life setting the groups were villages allocated to either treatment (either one of the OSP planting material delivery models) or control (they didn’t receive OSP planting material). Within these villages households were recruited to receive planting material (treatments) or not(control) and data on production, consumption and intake as well as biomarkers from target populations within households (women and children) were collected at the baseline, i.e., before the OSP planting material delivery interventions, and at the endline, two years after the interventions. Between the delivery intervention and endline, households made a series of decisions on whether or not to grow the planting material, what area, what to do with the harvest (household consumption vs sales), how to process and who in the household to consume and how much. The interventions did provide information on the best practices for production, processing, consumption to get the best outcomes, as well as nutrition related information, but the decisions at every turn were the households’ (De Brauw et al., 2018).

**Figure 8:**
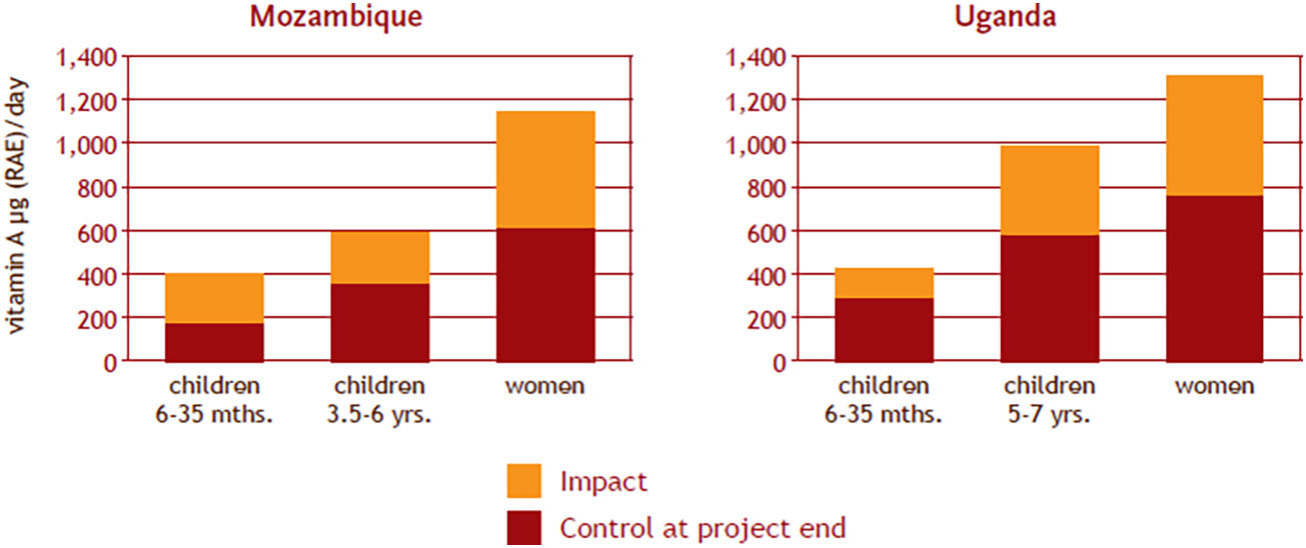
Impact of OSP delivery interventions on mean vitamin A intakes.

Evidence from Uganda showed that delivery of OSP resulted in significantly increased vitamin A intakes among children and women, and measurably improved vitamin A status among some children (Hotz et al., 2012a). In Mozambique, delivery of OSP resulted in increased vitamin A intakes, in fact doubling of vitamin A intakes among households who grew OSP, with OSP providing almost all of the total vitamin A intakes for children (Hotz et al., 2012b) (**Figure 8**). These effectiveness studies also showed that the cheaper, less intensive delivery model was as effective as the more expensive and more intensive delivery model in reaching these outcomes. The most effective-least cost delivery model tested cost USD15–20 per DALY saved, which is considered highly cost-effective by World Bank standards (World Bank, 1993; Arimond et al., 2010). Based on these results, significant funding from USAID – FTF for Uganda facilitated the scaling of the less intensive model to benefit many more Ugandan households (Foley et al., 2021, Uganda Outcome Impact Case Report).

Further analysis of this effectiveness study data from Mozambique revealed that regular consumption of OSP also reduced child morbidity: in children under five, the likelihood of experiencing diarrhea was reduced by 39 percent, and duration of diarrhea episodes was reduced by more than 10 percent; in children under three, the reductions were by 52 percent and 27 percent, respectively (Jones and de Brauw, 2015). A follow up study conducted three years after the endline of the Mozambique study found that vitamin A intakes remained higher among women in the intervention households and their young children born after the trial—demonstrating the long-term adoption and sustainability of biofortification as a food-based intervention (De Brauw et al., 2019).

According to Lancet Maternal and Child Health Nutrition Series (2013) “The feasibility and effectiveness of biofortified vitamin A-rich orange sweet potato for increasing maternal and child vitamin A intake and status has been shown”, and “Evidence of the effectiveness of targeted agricultural programmes on maternal and child nutrition, with the exception of vitamin A, is limited…. and rigorous effectiveness assessments are needed.” (Black et al., 2013).

At the beginning of the program, the intention of socio-economic and nutrition teams was to conduct an effectiveness study for each one of the seven key biofortified crops (vitamin A cassava, maize and OSP; iron beans and pearl millet; zinc rice and wheat). Given the time and the resources needed to conduct such studies, favorable results of nutritional efficacy studies, and increasing availability of program monitoring data and modelling tools, HarvestPlus socio-economics and nutrition teams, in consultation with program leadership and key donors, agreed to conduct only two additional effectiveness studies – one for each micronutrient. In addition to OSP in Mozambique (which covered a vitamin A intervention, a vegetatively propagated crop, an informal seed system and the Africa region) two other studies were proposed. One for iron beans in Guatemala (covering an iron intervention, a legume crop in a semi-formal seed system, and the LAC region) and the other for zinc wheat in Pakistan (covering a zinc intervention, a cereal crop in a formal seed system and the Asia region). Guatemala iron bean intervention study was implemented 2014-2019, the trial running longer than expected due to several seasons of droughts that required re-delivery of iron bean seed, as well as difficulty in following up with the target household members (adolescent girls many of whom aged or moved out/got married) whose iron status were being monitored. By the end of 2021 the analysis of the data was completed and a paper is forthcoming. The effectiveness study for zinc wheat in Pakistan will be implemented once cost-effective and practical zinc biomarkers sensitive enough for detecting changes in zinc status as a result of changes in dietary zinc intake are identified (Knez et al., 2022).

As a final note, the impact indicators listed under the MEL indicators in **Table 1**, are estimated by using a tracking and forecasting model, which combines secondary data on the national level demographics, production and consumption of the crop, and health statistics with program level data on output and outcome indicators collected and program costs recorded. These models help estimate the three impact indicators which are the estimated average requirement (EAR) of micronutrient delivered through biofortification of the staple with that micronutrient; change in the prevalence of inadequate intake of target micronutrient as a result of the biofortification program, and the number of DALYs averted as a result of biofortification, as well as the cost per DALY saved (and program benefit cost ratio or return on investment [ROI]).

A recent example for such analysis is that for Rwanda which found that as a result of the iron bean biofortification program implemented in that country from 2012-2018, overall USD 5 million worth of DALYs were saved that would have been lost to iron deficiency. Combined with the USD20 million worth of improved yields as a result of farmers growing seeds of improved varieties, the total benefits of the program for 2012-2018 amounted to USD25 million, as compared with the total costs of the program during that period (USD10M), revealing a 1:2.5 RoI during the program period. The HarvestPlus program and expenditures in Rwanda ended in 2018, but the benefits of continued adoption, continue to accrue over time (HarvestPlus, 2019).

## 4 Discussion

For an intervention that sits squarely at the intersection of agriculture and nutrition, the role of socio-economic research in biofortification may not be obvious. After all, plant breeders are needed to develop biofortified varieties and nutritionists are needed to assess if these biofortified varieties do indeed improve nutrition outcomes. A number of varied actors and institutions are required for scale up of biofortified varieties, as biofortification sits at the nexus of agriculture and nutrition, just as these two disciplines also sit at the nexus of dynamic human interactions and global development. As such a key question is how can socio-economists add value to this mix?

During its first 20 years of operation, HarvestPlus will have invested approximately $500 million in its various activities. Justifying and motivating overall spending and allocating resources within the program requires understanding of the trade-offs among investments; identification of opportunities and challenges and their effects in achieving program objectives; observation of the choices made by the agents of change – from farmers to processors to consumers to policy-makers and investors and how they can be “nudged” in favor of biofortification. This is where socio-economic research comes in.

**Figure 9:**
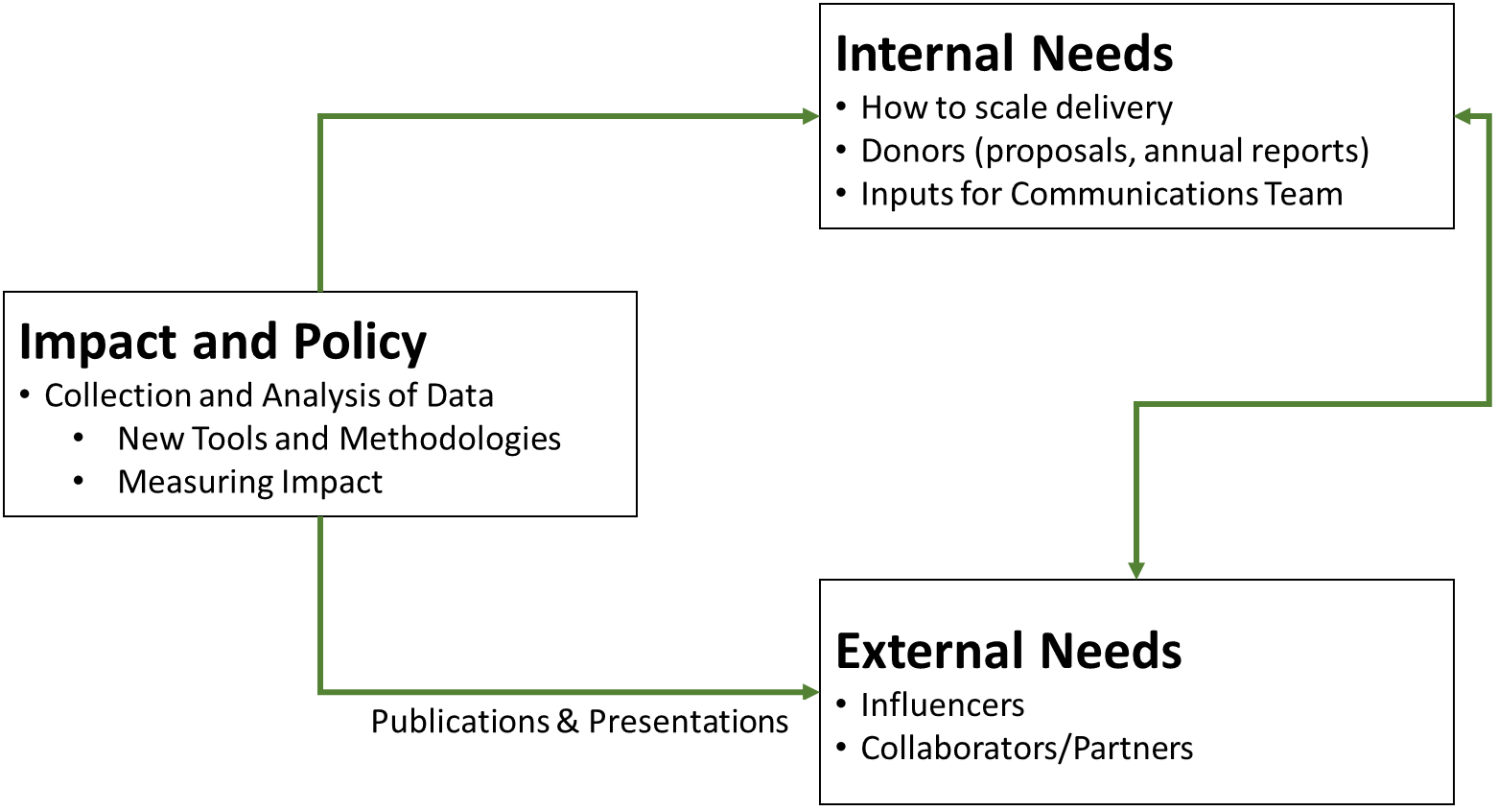
Roles and responsibilities of the socio-economics team at HarvestPlus.

As **Figure 9** shows, informational needs generated by socio-economists are both internal to the program and external. Before varieties were available for scale up, *ex ante* economic analysis showed the potential value of biofortified crops, information which was essential for securing long-term funding. Once biofortified crops were released and subsequently delivered, information generated by socio-economists was used internally to guide and improve the efficiency of scaling strategies. Data were collected on the reach (households growing) and impact of biofortified crops (as shown by effectiveness and impact assessment studies). Analysis of these data were published externally, to influence national and international policies and programs, private seed and food companies, as well as humanitarian organizations to integrate biofortification where staples are concerned, and until such time comes that biofortified staples become the new normal, to secure donor funding for HarvestPlus to continue catalyzing scaling of biofortification.

This paper has presented the key pillars of the portfolio of socio-economics research conducted for the CGIAR biofortification program, HarvestPlus. It provides an overview of type of socio-economic research that can be conducted from the beginning (e.g., *ex ante* impact and cost-effectiveness analyses to attract investment for test of concept) to the end (e.g., codified learnings and easy-to-use tools to inform scaling of interventions) for any new improved variety of crops, or in fact for any new technology.

Just as production of biofortified crops and their consumption as food go to scale, socio-economic research on biofortification should also do the same. After two decades of HarvestPlus investments in socio-economics research for biofortification, it is now time for the One CGIAR, as well as universities and research centers around the world to continue to implement and grow this portfolio. There are still many activities to be undertaken and questions that need investigating by using socio-economic tools and methods, for example: what is the impact of climate change on biofortified crop yield and nutrient content on farm, and to what extent can biofortification mitigate the negative impact of climate change on food and nutrition insecurity?; what are the most cost-effective and inclusive mix of micronutrient interventions (biofortification, supplementation, commercial fortification and others) temporally and spatially?; what is the role of biofortified crops and foods in humanitarian programs?; how can biofortification programs be sustainable and result in equitable outcomes?; how could technological advances, such as gene editing or artificial intelligence and augmented reality, be useful improving breeding and delivery programs?; what is the value of implementing MEL at scale and having a global monitoring system to track the coverage and impact of biofortification, and continued assessment of the acceptance and effectiveness of new biofortified crops and their varieties as they are developed.

Overall, awareness and efforts to link agri-food systems to human nutrition are more in evidence now compared to 20 years ago when biofortification efforts had started. It is however uncertain how this will play out and be sustained. It is important to show successes, not just overall impact but distribution thereof, but impacts of agri-food system interventions develop slowly. Biofortification is in the forefront of demonstrating just how resilient, sustainable, and cost-effective such interventions can be for improving nutrition and health through agri-food systems. Although substantial progress has been made, biofortification is not yet tightly woven into the fabric of present-day agri-food systems – from research to table. Socio-economics research including – but not limited to - what is presented in this paper, is instrumental in ensuring that the full potential of crop development technologies, such as biofortification can be realized.

## Author contributions

All authors listed have made a substantial, direct, and intellectual contribution to the work and approved it for publication.
